# Gibberellins in developing wheat grains and their relationship to late maturity α-amylase (LMA)

**DOI:** 10.1007/s00425-022-03899-y

**Published:** 2022-05-06

**Authors:** Daryl Mares, Adinda Derkx, Judy Cheong, Irina Zaharia, Robert Asenstorfer, Kolumbina Mrva

**Affiliations:** 1 School of Agriculture, Food and Wine, University of Adelaide, Waite Campus, Glen Osmond, SA 5064 Australia; 2 South Australian Agricultural Research Institute, Waite Precinct, Glen Osmond, SA Australia; 3 National Research Council of Canada, Government of Canada, 110 Gymnasium Place Saskatoon, Saskatchewan, S7N 0W9 Canada

**Keywords:** Aleurone, α-Amylase protein, GA biosynthesis inhibitors, Gene transcripts, Plant hormones

## Abstract

**Main conclusion:**

α-Amylase synthesis by wheat aleurone during grain development (late maturity α-amylase) appears to be independent of gibberellin unlike α-amylase synthesis by aleurone during germination or following treatment with exogenous GA.

**Abstract:**

Late-maturity α-amylase (LMA) in wheat (*Triticum aestivum* L.) involves the synthesis of α-amylase by the aleurone tissue during grain development. Previous research identified a putative *ent*-copalyl diphosphate synthase gene, coding for an enzyme that controls the first step in gibberellin biosynthesis, that underlies the major genetic locus involved in variation in LMA phenotype. The reported results for gene transcript analysis, preliminary gibberellin analysis and the effects of DELLA mutants on LMA phenotype appeared to be consistent with involvement of gibberellin but did not provide definitive proof of a causal link. Conversely, several observations do not appear to be consistent with this hypothesis. In this current study, LMA phenotype, gibberellin profiles and ABA content were recorded for experiments involving susceptible and resistant genotypes, gibberellin biosynthesis inhibitors, genetic lines containing different LMA quantitative trait loci and treatment of distal halves of developing grains with exogenous gibberellin. The results suggested that gibberellin may not be a prerequisite for LMA expression and further that the mechanism involved in triggering α-amylase synthesis did not correspond to the model proposed for germination and gibberellin challenged aleurone of ripe grain. The results provide new insight into LMA and highlight the need to investigate alternate pathways for the induction of α-amylase gene transcription, the function of novel 1-β-OH gibberellins and other functions of DELLA proteins in developing grains.

**Supplementary Information:**

The online version contains supplementary material available at 10.1007/s00425-022-03899-y.

## Introduction

Developing grains of wheat may synthesise high pI α-amylase, TaAmy1, during the middle stages of the interval between anthesis and harvest-ripeness depending on the genotype and the environment. Once formed, the enzyme is retained until harvest and can result in a falling number that is below receival standards. This phenomenon is referred to as late maturity α-amylase (LMA) or pre-maturity α-amylase (PMA) and it represents a significant constraint for wheat breeders in many parts of the world.

Mares and Gale (1990) used isoelectric focussing (IEF) to demonstrate that the endosperm of LMA-affected grain contained 4 or 5 bands of high pI α-amylase, TaAmy1, but no low pI α-amylase, TaAmy2. The latter group of isozymes that were clearly separated from the high pI bands on the IEF gels, were detected in the pericarp during the first 10–15 days after anthesis (DPA) but then disappeared as the grains matured. The high pI bands in LMA-affected grain were also present in grain during the early stages of germination. Earlier research by Gale et al. (1983) identified 16 bands of low pI α-amylase with isoelectric points at pH between 4.8 and 5.7 in extracts from GA_3_-treated distal sections of grain of Chinese Spring. The same extracts contained bands of high pI α-amylase with isoelectric point between pH 6.6 and 7.5. Cheng et al. (2014) analysed the *TaAmy1* gene family members expressed in developing grain of LMA-susceptible wheat grains. Five gene sequences showed a high frequency of expression based on sequencing 91 clones in an LMA-susceptible line. Five high isoelectric point groups were predicted based on the translated partial coding sequences. Cheng et al. (2014) reported that it appeared that protein sequences with the same isoelectric point could be encoded by different chromosome 6 sub-genomes. This would explain why Gale et al. (1983) were unable to map all isozymes with a specific isoelectric point to a particular chromosome. *TaAmy1* genes were expressed in developing grain of LMA-susceptible but not in LMA-resistant lines (Barrero et al. 2013). Neither *TaAmy2* nor *TaAmy3* were expressed, whilst *TaAmy4* was expressed at low levels in both susceptible and resistant lines. Bread wheat contains four families of α-amylase genes, *TaAmy1*, *TaAmy2*, *TaAmy3* and *TaAmy4*, that are expressed at various stages in grain development and germination (Mieog et al. 2017). Mieog et al. (2017) suggested the potential involvement of TaAmy4 in the LMA phenotype, however the results of a subsequent study appear to confirm that most of the α-amylase activity is generated by TaAmy1 (Newberry et al. 2018). Whilst a role for TaAmy4 cannot be excluded, the mechanism through which TaAmy4 could be involved remains to be elucidated.

The expression of LMA is strongly regulated by temperature which creates a distinction between tall wheat varieties and semi-dwarf varieties based on gibberellin insensitivity genes. Whereas tall varieties that are LMA-susceptible respond under a wide range of temperatures, LMA-susceptible semi-dwarfs appear resistant at warmer temperatures but can be induced to synthesise α-amylase either by applying a cool temperature shock during grain development (Mrva and Mares 2001a; Mrva et al. 2006; Farrell and Kettlewell 2008; Mares and Mrva 2014; Derkx and Mares 2020) or by maintaining the daily maximum temperature during grain development below around 23 °C (Derkx and Mares 2020).

Whilst there has been considerable progress in understanding the genetic and environmental factors involved in LMA (Lunn et al. 2001; Mrva and Mares 2001a; Mares and Mrva 2014; Armstrong et al. 2020; Derkx and Mares 2020; Derkx et al. 2021; Liu et al. 2021), the biochemical mechanisms that lead to the coordinated synthesis of high pI α-amylases coded by the *TaAmy1* genes on the long arms of the group 6 chromosomes are not well understood. In some respects, α-amylase synthesis associated with LMA is similar to that associated with grain germination. Both result in the appearance of high pI α-amylase that is transported from the site of synthesis into the starchy endosperm. However, during germination, the enzyme is initially synthesised in the scutellum and only later in the aleurone in response to gibberellin, GA, produced by and transported from the embryo (Lenton and Appleford 1993). As a consequence, α-amylase is initially concentrated at the proximal or embryo end of the grain and spreads towards the distal end of the grain as germination progresses resulting in a marked concentration gradient. By comparison, LMA affected grain does not show such a gradient, rather there is a similar amount of α-amylase in both the proximal and distal half of the grain (Mrva et al. 2006). In addition, there is no evidence that the embryo is involved in the LMA response. Rather, the report by Derkx et al. (2021) suggests that synthesis of GA in developing grain takes place in the aleurone. Leaving aside the α-amylase synthesis that takes place in the scutellum of the germinating grain, it is clear that in both LMA and germination there is α-amylase synthesis in aleurone tissue. This raises the question of whether the mechanism involved in LMA is similar to the well-established GA-stimulated synthesis of α-amylase by the aleurone in germinating grains or in GA-challenged aleurone of de-embryonated barley grains described by Gubler et al. (1995). Subsequent studies have proposed that GA combines with DELLA, a negative regulator of GA signalling, and the gibberellin receptor GID1 to facilitate the degradation of the DELLA protein resulting in the activation of the GAMyb transcription factor and α-amylase production (Fu et al. 2002; Gubler et al. 2002; Zantella et al. 2002).

The observed reduction in LMA in semi-dwarf wheat varieties based on DELLA mutants, *Rht-B1b*, *Rht-D1b*, and *Rht-B1c*, that are associated with insensitivity to gibberellin (Mrva and Mares 1996), but not tall varieties or varieties based on alternate semi-dwarfing genes such as *Rht8* (Mrva et al. 2008), would appear to support a similar role for GA in LMA (Derkx et al. 2021). Although why this only applies under warmer temperatures requires an explanation. Kondhare et al. (2012) applied GA_3_ and ABA, separately and in combination, in situ to intact developing grains during mid-grain development then measured total α-amylase at maturity. Application of GA_3_ significantly increased α-amylase in the cultivar Rialto (high LMA susceptible) but not in Spark (low LMA susceptible). In addition, spikes were harvested from plants at a later stage of development and embryoless half-grains incubated in hormone solution prior to measurement of α-amylase activity. In this in vitro experiment, GA_3_ increased α-amylase activity in both Rialto and Spark. The authors concluded that GA-response was a major factor during LMA induction whereas ABA-response appeared to be of less importance. Subsequently, Kondhare et al. (2013) investigated the role of sensitivity to gibberellin and abscisic acid in pre-maturity α-amylase formation. The results suggested that LMA was related to an increase in GA-sensitivity of the aleurone at around 640° DPA. Kondhare et al. (2014a) followed up these investigations and used hormone biosynthesis inhibitors to determine the effects of altered ABA and GA levels on LMA formation. They reported that the results indicated an association between GA levels at mid-grain development and PMA formation. A more recent study by Barrero et al. (2013) showed that LMA was associated with an increase in GA precursor accumulation. Neither GA_1_ nor GA_4_ was reported, however, it is possible that they were present but below the level of detection.

There are, however, characteristics of LMA and developing grains that do not appear to be consistent with the model proposed for α-amylase synthesis by the aleurone of ripe grain during germination. LMA seems to involve only the high pI *αAmy1* genes and not the other GA-inducible genes typical of the GA response of mature aleurone (Barrero et al. 2013) despite the genes having the same or similar GAMyb binding domain (Gubler et al. 1999), a GA-responsive promotor element called a G-box. Barrero et al. (2013) concluded that the LMA phenotype seemed to be a partial or incomplete gibberellin response. Other studies (Wheeler, 1972; Radley 1976) have shown that GA bioactivity peaks during early grain development and declines well before the aleurone becomes responsive to exogenous GA_3_ late in ripening when grain moisture has decreased to less than 25% (Armstrong et al. 1982). In addition, developing wheat grains synthesise 1-β-hydroxy GAs such as GA_54_ and GA_55_ (Gaskin et al. 1980; Lenton and Gale 1987; Pearce et al. 2015), rather than the more common GA_1_, GA_3_ and GA_4_ found in germinating grains (Lenton and Appleford 1991; Appleford and Lenton 1997). Pearce et al. (2015) reported that *TaGA1ox1* and *TaGA3ox3*, genes that code for the GA 1-oxidase and GA 3-oxidase specific to their model for the synthesis of GA_54_, were highly expressed in developing wheat grains. *TaGA20ox3*, that codes for the GA20 oxidase required to generate GA_9_, the substrate for GA 1-oxidase, was also highly expressed in developing grain. Lenton and Gale (1987) had earlier proposed that both GA_54_ and GA_55_ could be derived by sequential 1-hydroxylation and 3-hydroxylation reactions starting with GA_9_ and GA_20_, respectively. These novel GAs were biologically active albeit with 30–100 times lower activity compared with GA_1_ in a barley endosperm assay (Lenton and Gale 1987) and are not present in harvest-ripe or germinating wheat grains (Lenton and Appleford 1991).

Several quantitative trait loci (QTL) have been reported to be associated with variation in the LMA phenotype in wheat (Mrva and Mares 2001b; Mrva et al. 2009; Emebiri et al. 2010; Tan et al. 2010; Derkx et al. 2021). Of the QTL that have been identified, one located towards the distal end of the long arm of chromosome 7B has the most significant impact on LMA phenotype, explaining up to 52% of the observed variation in populations derived from crosses between resistant and susceptible lines (Derkx et al. 2021). Derkx et al. (2021) reported that the causal gene associated with the 7B QTL is a putative *ent*-copalyl diphosphate synthase (CPS) which, based on protein sequence comparison with previously published *ent*-CPS (Wu et al. 2012), appears to be a novel member of the CPS family in wheat. Single nucleotide polymorphisms (SNPs) were identified by comparing the nucleotide sequence of the coding region of the gene from around 50 wheat lines. Further, KASP markers based on these SNPs were used to screen a large panel of varieties that were subsequently assigned to nine haplotype groups (Derkx et al. 2021). The CPS gene was expressed in the endosperm prior to the appearance of α-amylase protein and transcript abundance was very low in the haplotype groups that contained known resistant lines, Chinese Spring, Maringa or Halberd. Within the other haplotype groups there did not appear to be a significant correlation between LMA phenotype and transcript abundance.

Interestingly, *ent*-copalyl diphosphate synthase together with another terpene cyclase, *ent*-kaurene synthase, catalyse the initial cyclisation of geranylgeranyl diphosphate, the first step in the biosynthetic pathway for gibberellins (Spielmeyer et al., 2004) An earlier study (Barrero et al. 2013) reported that concentrations of GA pathway intermediates such as GA_19_ were greatly reduced in de-embryonated developing grains of the resistant variety, Maringa, as well as in lines from a Spica (LMA-susceptible)/Maringa (LMA-resistant) population that carried Maringa alleles at the 7B locus. This observation has served to highlight the need for more research into the link between LMA and temporal changes in plant hormone content of developing wheat grains and to extend the work to a greater number of varieties. Of particular interest in this context would be varieties that have been shown to have a functional CPS gene that is transcribed during grain development but retain a resistant phenotype (Derkx et al. 2021).

The aims of this investigation were to clarify the role of gibberellin in the expression of LMA and provide new information on the mechanism involved in the synthesis of high pI α-amylase in developing wheat grains.

## Materials and methods

### Germplasm and plant growth conditions

Seed of Australian varieties, Spica (Three-Seas/Kambourica//Pusa-4/Flora–released in 1952) and Hartog (Vicam-71//Ciano-67(sib)/Siete Cerros-66/3/Kalyansona/Bluebird–released in 1982), the breeding line RAC655 (CHA/Mengavi-8156//Ciano-67(sib)/Gallo//Bezostaya-2(CO-2224)/3/RAC-309-S–breeding line from late 1990s not released commercially due to a high LMA rating) and the Brazilian variety, Maringa (Frontana/Kenya-58//Ponta-Grossa-1—released in 1966), were obtained from stocks maintained at the University of Adelaide. These varieties maintained a consistent LMA phenotype over many experiments × seasons and with the exception of the red wheat, Maringa, are hard-grained, white spring wheats with no vernalisation or photoperiod requirement. Spica (LMA-susceptible)/Maringa (LMA-resistant) doubled haploid lines have been previously described in Barrero et al. (2013) and Derkx et al. (2021). Reciprocal F_1_ grains were prepared by pollination of emasculated spikes of Spica (LMA-susceptible) and Maringa (LMA-resistant) with pollen from the alternate parent. Semi-dwarf varieties Hartog (LMA-resistant) and RAC655 (LMA-susceptible), represent lines that carry an LMA-susceptible allele at the major LMA 7B locus but have resistant and susceptible LMA phenotypes, respectively (Derkx et al. 2021). Both Hartog and RAC655 are included in the panel of LMA standards that is used in Australia to determine LMA risk, a process that is a prerequisite for grade classification and variety release.

Wheat lines were grown in a standard glasshouse equipped with evaporative air-conditioning but where temperature varied due to differences in daily solar radiation. Mean maximum temperatures in the glasshouse for the period anthesis to maturity were typically between 25 and 30 °C. For cool shock treatments, tillers were transferred to a controlled environment room with temperatures set at 18 °C during the 14 h light period and 12 °C during the dark (Derkx and Mares 2020). Plants were grown in 20 cm diameter pots with four plants per pot. Tillers were tagged at anthesis (first anthers extruded; Zadoks growth stage 61) and sampled at intervals post-anthesis, depending on the particular experiment, and then at harvest-ripeness (grain moisture of around 12% FW) which was reached at 50–55 DPA.

### Chemicals and antibodies

Polyclonal and monoclonal antibodies required for LMA ELISA were covered by a sub-licence to the University of Adelaide by the Grains Research and Development Corporation, Australia, and were provided from stocks maintained by the South Australian Research and Development Institute, Urrbrae, South Australia. Labelled (horseradish peroxidase, HRP) anti-mouse antibody was sourced from Merck Australia, while the substrate for HRP, Blue Substrate ESBP1000, was obtained from ELISA Systems, Queensland, Australia. Other chemicals used in the study were obtained from Sigma-Aldrich (Macquarie Park, NSW, Australia).

### Determination of high pI α-amylase protein content and total α-amylase activity

De-embryonated grain was milled into a fine meal using a Perten LM-3310 laboratory burr mill equipped with type-2 fine grinding discs (Perten Instruments, Macquarie Park, NSW, Australia).

High pI α-amylase protein content. Four 100 mg technical replicates per grain sample were assessed for high pI α-amylase content using a sandwich ELISA assay developed by Verity et al. (1999) and the protocol described in Barrero et al. (2013) adapted to a 96-well format with C96 Maxisorb Nunc-Immuno microplates (Nunc A/S, Roskilde, Denmark). The second antibody used in the sandwich ELISA bound the high pI α-amylase (Verity et al. 1999) but not low pI α-amylase. Spectrometric measurements were performed using a Benchmark™ Plus microplate reader (Bio-Rad Laboratories, Hercules, CA, USA) at 595 nm, and results were expressed in optical density (OD) units.

Total α-amylase activity was determined on the meal samples in triplicate using a modification of the Megazyme Amylazyme™ (Megazyme Ltd., Bray, Ireland) assay. Following the assay which was run according to a modification of the manufacturer’s instructions, rather than using 1 cm pathlength cells in a spectrophotometer, 100 µL aliquots of the centrifuged reaction mixture supernatant were transferred to each of 4 wells on a 96 well microplate (Nunc TC microwell 96F Nunclon D microplate, 6 mm well diameter, Nunc A/S) and OD measured at 590 nm in a Bio-Rad Benchmark Plus microplate reader (Bio-Rad Laboratories). Results were expressed as optical density units. Microplate readings were strongly correlated with spectrophotometer readings (*R*^2^ = 0.995).

### Extraction and quantitation of gibberellins and ABA

Developing grains were collected from floret positions 1 and 2 of the central part of spikes sampled at intervals post-anthesis (Supplementary Fig. S1). Triplicate samples from each of 3 or more spikes were collected at each sampling time unless stated otherwise. The embryo end of the grains was removed with a scalpel and the de-embryonated grain samples were frozen in liquid nitrogen, freeze-dried and milled into a fine meal with a RotoMix™ capsule mixing unit (3 M ESPE, St. Paul, MN, USA) for 7 s using a 4 mm stainless steel ball-bearing. Gibberellin and ABA profiling was carried out at the Aquatic and Crop Resource Development Research Centre of the National Research Council Canada) (https://nrc.canada.ca/en/research-development/products-services/technical-advisory-services/plant-hormone-profiling) using a modified procedure described in Lulsdorf et al. (2012). Briefly, analysis was performed on a UPLC/ESI–MS/MS utilizing a waters ACQUITY UPLC system, equipped with a binary solvent delivery manager and a sample manager coupled to a Waters Micromass Quattro Premier XE quadrupole tandem mass spectrometer via a Z-spray interface. MassLynx™ and QuanLynx™ (Micromass, Manchester, UK) were used for data acquisition and data analysis.

### Application of exogenous hormones and gibberellin biosynthesis inhibitors

Sister lines Sp/M52 (LMA-susceptible) and Sp/M47 (LMA-resistant) used in this experiment reached anthesis within 5 days of each other whilst both the time from anthesis to grain ripeness and the time course of grain moisture loss during ripening were not significantly different. GA_3_, ABA, paclobutrazol or prohexadione calcium, 50 µM in de-ionised water, were injected into the hollow stem of the peduncles (0.6–0.9 mL depending on the length and diameter of the peduncle) of tagged spikes at 10 DPA. For each line, only spikes that reached anthesis on the same day were used. In addition, plants were sprayed with 20 µM unbuffered solutions of these compounds containing a small amount of Tween 20 on the same day that hormone was injected into the peduncle. Tween 20, 2 drops per 100 mL hormone solution, was added as a surfactant to aid wetting and absorption by the leaves and vegetative parts of the spikes.

### Response of de-embryonated grain to exogenous gibberellin

Triplicate batches of 20 grains from Spica, Maringa, Sp/M52, Sp/M47, RAC655 and Hartog were sampled at intervals from 20 DPA and de-embryonated sections incubated on filter paper moistened with 20 µM GA_3_ for 3 days at 20 °C. Following the incubation, the grains were frozen in liquid nitrogen and freeze-dried. The samples were reduced to a fine meal in a RotoMix™ capsule mixing unit (3 M ESPE) as described above. Total α-amylase activity was determined using a modification of the Megazyme Amylazyme™ (Megazyme Ltd) assay as described above.

### Synthesis and determination of GA_55_

Gaskin et al. (1980) reported the presence of 1-β-hydroxyl gibberellins, GA_54_ and GA_55_, in developing grain of the wheat variety Maris Huntsman. Before this report, Murofushi et al. (1979) had described the isolation and synthesis of these GAs. Since authentic standards were not commercially available for GA_55_ and GA_54_, their presence could not be determined by the Canadian plant hormone analytical service described above.

Synthesis of GA_55_ was achieved using commercially available GA_3_ as the starting material via a 2-step procedure; the first step, a Birch reduction using lithium as the metal catalyst (Shimano et al. 1990) resulted in the production of GA_3_H_2_. This product was then oxidised in a second step with *m*-chloroperoxybenzoic acid (Murofushi et al. 1979) to yield GA_55_. Mass spectrometry yielded two compounds with mass equivalent to GA_55_ (MH^+^ 363.15) indicating a mixture of 1,3-cis (75%) and 1,3-*trans* (25%) isomers. Pure 1,3-*trans* GA_55_ was isolated from the reaction mix by silica gel thin-layer chromatography (ethyl acetate (20); chloroform (8); acetic acid (8); Rf 0.28, detected with iodine) and used for NMR characterisation (Supplementary Table S1). Standard curves were developed for 3-day and 4-day incubation periods at 20 °C for both GA_3_ and GA_55_ with distal halves of mature wheat grains, cv Hartog (Supplementary Fig. S2). α-Amylase activity was determined following the incubation using the Megazyme Amylazyme™ assay described above. The response curves were used to estimate the biological activity of GA_55_ relative to GA_3_. On a mass (µg/mL) basis and absorbance of 1, GA_3_ was 50 times more active than GA_55_ for the 3-day bioassay and 190 times more active for the 4-day bioassay.

Extraction and quantitation of GA_55_ from grain samples was carried out at the Australian Wine Research Institute, Metabolomics Australia, Waite Precinct, Urrbrae, South Australia, Australia. Gibberellins were extracted and quantified using the methods reported by Chiwocha et al. (2003, 2005). The quantitation analysis of GA_55_ was performed on an Agilent 1290SL HPLC coupled to a QQQ 6490A. Samples were acquired in Multiple Reaction Monitoring (MRM) mode using negative ionization and optimized mass parameters for the compound of interest. The column used was a Kinetex PFP 2.6 µm, 150 × 210 mm and the HPLC solvents used were 10 mM ammonium formate in Milli-Q Water (solvent A) and methanol (solvent B) based on Urbanova et al. (2013). The data processing for the GA_55_ calibration curve and test samples were carried out using Agilent MassHunter (v A.00.06.36) software. GA_55_ calibration parameters include a limit of detection of 2 µg/L, a limit for quantitation of 5 µg/L, a linear calibration range of 2–500 µg/L and a *R*^2^ of 0.999.

### RNA isolation and gene expression

Transcript abundance of GA 1-oxidase, *TaGA1 ox1*, GA 3-oxidase, *TaGA3 ox3*, GA 20-oxidase (*Ta GA20 ox3*) genes that code for the enzymes required for the synthesis of 1-β-hydroxy gibberellins, was determined using three biological replicates each consisting of 20 seeds collected at 10, 15, 18, 21, 24, 27 or 30 DPA from plants grown in the glasshouse. After removing the embryo section, the de-embryonated seeds were crushed in liquid nitrogen using a mortar and pestle. RNA preparation and qRT-PCR were as described in Derkx et al. (2021) and fold differences in transcript for the genes were calculated as relative fold increase using the PFAFFL method (Pfaffl 2001) and control transcript Actin. Primers for GA1ox1, AGTAGGGCCGCTTAAGGAAG forward and GGTTGATGGAGACCATCTCG reverse, GA3ox3, GTGATGCAGAGCCACGTC forward and TGAGGATCTGGAAGAGGTCA reverse, GA20ox3, ACCGTGTCCTTCAACTGCTC forward and CCCATGTCACGGTACTCCTC reverse, were from Pearce et al. (2015).

### Statistical analysis

Standard errors were calculated in MS Excel. Statistical differences between samples were determined by ANOVA (Genstat 19th Edition, www.vsni.com.uk) at a Bonferroni corrected significance level of 0.05.

## Results

### Hormone concentrations in developing grains of an LMA and a non-LMA genotype

GA_1_, GA_3_, GA_4_, GA_7_, GA_8_, GA_9_, GA_19_, GA_20_, GA_24_, GA_29_, GA_34_, GA_44_, GA_51_, and GA_53_ were assayed at 5-day intervals from 15 to 50 DPA along with several ABA-related compounds (neo-phaseic acid, 79-hydroxy-ABA, phaseic acid, dihydrophaseic acid and cis- and trans-ABA). None of the biologically active gibberellins, GA_1_, GA_3_ or GA_4_, were detected in grains at any stage between 15 and 50 DPA. Harvest-ripeness, 12% grain moisture, was attained between 55 and 60 DPA. Low concentrations of the biologically inactive precursor, GA_19_, were present in the grain of the LMA-susceptible genotype, Sp/M52, at 15 DPA (Table 1) and subsequently, GA_19_ and GA_44_ increased to peak around 30–35 DPA before declining to low concentrations by 50 DPA (Table 1). These intermediates are produced by the 13-hydroxy gibberellin branch of the GA biosynthesis pathway that leads to the formation of GA_1_ and/or GA_55_ (Supplementary Fig. S3). Intermediates present in the non-13 hydroxyl GA pathway were either not detected or were below the level required for quantitation as previously reported (Derkx et al. 2021). By comparison, in the LMA-resistant line, Sp/M47, concentrations of these gibberellins remained below the level required for quantitation throughout grain development and were significantly different from Sp/M52 (*P* = 0.05) at all sampling times. GA_8_, the inactive breakdown product of GA_1_, was either below the level of detection or was below the level required for quantitation. ABA concentrations were already high in both genotypes at 15 DPA, increased to a peak around 35–40 DPA before declining to lower concentrations by 50 DPA. Differences in ABA concentration between Sp/M52 and Sp/M47 were not significantly different (*P* = 0.05).

**Table 1:** Concentrations of gibberellins and ABA, together with high pI α-amylase protein (ELISA) and moisture content during grain development and ripening in an LMA and a non-LMA genotype

Genotype/treatment	DPA	GA_19_ ng gDW^−1^	GA_44_ ng gDW^−1^	ABA ng gDW^−1^	High pI α-amylase ELISA OD	Grain moisture % FW
Sp/M52/control	15	22 ± 4	nd	163 ± 6	0.11 ± 0.009	69 ± 0.1
Sp/M52/control	20	52 ± 4	15 ± 2	101 ± 6	0.19 ± 0.1	61 ± 0.3
Sp/M52/control	25	113 ± 11	37 ± 3	179 ± 10	**0.42 ± 0.06**	52 ± 1.3
Sp/M52/control	30	179 ± 26	51 ± 12	212 ± 39	**0.54 ± 0.02**	48 ± 0.6
Sp/M52/control	35	130 ± 10	77 ± 21	237 ± 33	**0.63 ± 0.01**	44 ± 0.8
Sp/M52/control	40	96 ± 7	65 ± 12	351 ± 54	**0.66 ± 0.01**	40 ± 0.5
Sp/M52/control	45	19 ± 3	nd	227 ± 57	**0.66 ± 0.005**	35 ± 1.7
Sp/M52/control	50	20 ± 4	nd	71 ± 32	**0.70 ± 0.006**	26 ± 6.5
Sp/M52/control	55	No data	No data	No data	**0.69 ± 0.008**	13 ± 1.2
Sp/M52/control	60	No data	No data	No data	**0.63 ± 0.01**	10 ± 0.5
Sp/M47/control	15	nd	nd	203 ± 9	0.08 ± 0.01	72 ± 0.8
Sp/M47/control	20	nd	nd	264 ± 53	0.09 ± 0.015	68 ± 1.5
Sp/M47/control	25	nd	nd	101 ± 8	0.08 ± 0.003	60 ± 0.2
Sp/M47/control	30	nd	nd	110 ± 4	0.09 ± 0.005	54 ± 0.4
Sp/M47/control	35	nd	nd	238 ± 22	0.09 ± 0.008	48 ± 1.9
Sp/M47/control	40	nd	nd	188 ± 15	0.12 ± 0.01	45 ± 0.6
Sp/M47/control	45	nd	nd	125 ± 10	0.15 ± 0.05	41 ± 0.7
Sp/M47/control	50	nd	nd	15 ± 2	0.10 ± 0.01	30 ± 2.3
Sp/M47/control	55	No data	No data	No data	0.11 ± 0.02	14 ± 0.2
Sp/M47/control	60	No data	No data	No data	0.11 ± 0.02	10.5 ± 0.5
Sp/M52 + paclo	25	*	nd	194 ± 30	**0.28 ± 0.015**	52 ± 2
Sp/M52 + paclo	30	*	nd	233 ± 24	**0.37 ± 0.03**	49 ± 0.3
Sp/M52 + paclo	35	*	nd	222 ± 25	**0.46 ± 0.01**	45 ± 0.3
Sp/M52 + paclo	40	*	nd	188 ± 18	**0.58 ± 0.01**	44 ± 0.7
Sp/M52 + paclo	60	No data	No data	No data	**0.56 ± 0.02**	11 ± 0.8
Sp/M52 + prohex	25	457 ± 43	434 ± 43	172 ± 14	0.08 ± 0.01	No data
Sp/M52 + prohex	30	404 ± 47	397 ± 45	169 ± 8	**0.26 ± 0.02**	No data
Sp/M52 + prohex	35	395 ± 14	400 ± 21	176 ± 6	**0.37 ± 0.02**	No data
Sp/M52 + prohex	40	323 ± 22	338 ± 23	159 ± 3	**0.48 ± 0.01**	No data
Sp/M52 + prohex	60	No data	No data	No data	**0.61 ± 0.01**	No data

Some plants of the LMA genotype, Sp/M52 selected from a Spica/Maringa population, were treated with gibberellin biosynthesis inhibitors, paclobutrazol (paclo) or prohexadione-Ca (prohex) at 10 DPA. All sampling was done in triplicate. Samples harvested at 55 and 60 DPA were not analysed for hormone concentration (no data) as this time in grain development was well past the period of α-amylase synthesis. These samples were analysed for high pI α-amylase protein (ELISA) and moisture content with the exception of the Sp/M52 + prohex where there was insufficient material for determination of grain moisture. Grain reached harvest-ripeness (≤ 12% moisture) between 55 and 60 DPA

*Signal below the level required for quantification, *nd* no signal detected, *DW* grain dry weight, *FW* grain fresh weight, standard errors are shown after the ± sign. Statistical significance was assessed with ANOVA using a Bonferroni corrected significance level of *P* = 0.05

High pI α-amylase-specific ELISA values shown in bold were associated with a strong colour change in the assay and indicate that α-amylase protein was present. Values in plain font were not associated with a colour change and indicate that α-amylase protein was absent or below the level of detection

High pI α-amylase protein ELISA OD increased in Sp/M52 from background levels (no detectable change in colour in the high pI α-amylase-specific ELISA) at 15–20 DPA to peak at 35–40 DPA and was retained through to ripeness at 55–60 DPA when grain moisture dropped to below 12% FW. From 25 DPA through to harvest-ripeness (HR), high pI α-amylase protein contents in Sp/M52 were significantly (*P* = 0.05) higher than Sp/M47. High pI α-amylase protein in the grain of the non-LMA genotype Sp/M47 remained at background level throughout grain development and ripening. Application of paclobutrazol to plants of Sp/M52 prior to 10 DPA appeared to inhibit the synthesis of GA_19_ and GA_44_ and concentrations of these metabolites remained below the level for quantitation throughout grain ripening. Interestingly however, high pI α-amylase protein content still increased, albeit more slowly than the untreated control, and by 35 DPA and through to harvest-ripeness was not significantly different (*P* = 0.05) from the non-treated control (Table 1). Paclobutrazol is a P450 monooxygenase inhibitor of *ent*-kaurene oxidase (EC 1.14.14.86) (Davidson et al. 2006), possibly other enzymes later in GA biosynthesis, and may affect other hormone biosynthesis pathways. *ent*-kaurine oxidase follows *ent*-copalyl diphosphate synthase (CPS: EC 5.5.1.12) and *ent*-kaurene synthase (EC 4.2.3.19) in the early stages of the gibberellin biosynthesis pathway (Supplementary Fig. S3). In this study, paclobutrazol did not appear to have a significant effect on ABA concentration (Table 1). Conversely, when plants of Sp/M52 were treated with prohexadione calcium, an inhibitor of 2-oxoglutarate-requiring dioxygenases that convert inactive to active gibberellins (Lenton and Appleford 1993), prior to 10 DPA there was a substantial and significant (*P* = 0.05) increase in concentrations of GA_19_ and GA_44_ compared with the non-treated control (Table 1). In addition, low concentrations of GA_20_, GA_24_, GA_29_ and GA_53_ were detected (Supplementary Table S2). Whilst there appeared to be a slower initial increase in high pI α-amylase protein, by 35 DPA and through to harvest-ripeness, the α-amylase protein content was not significantly different from the non-treated control or the samples from paclobutrazol-treated plants.

**Fig. 1:**
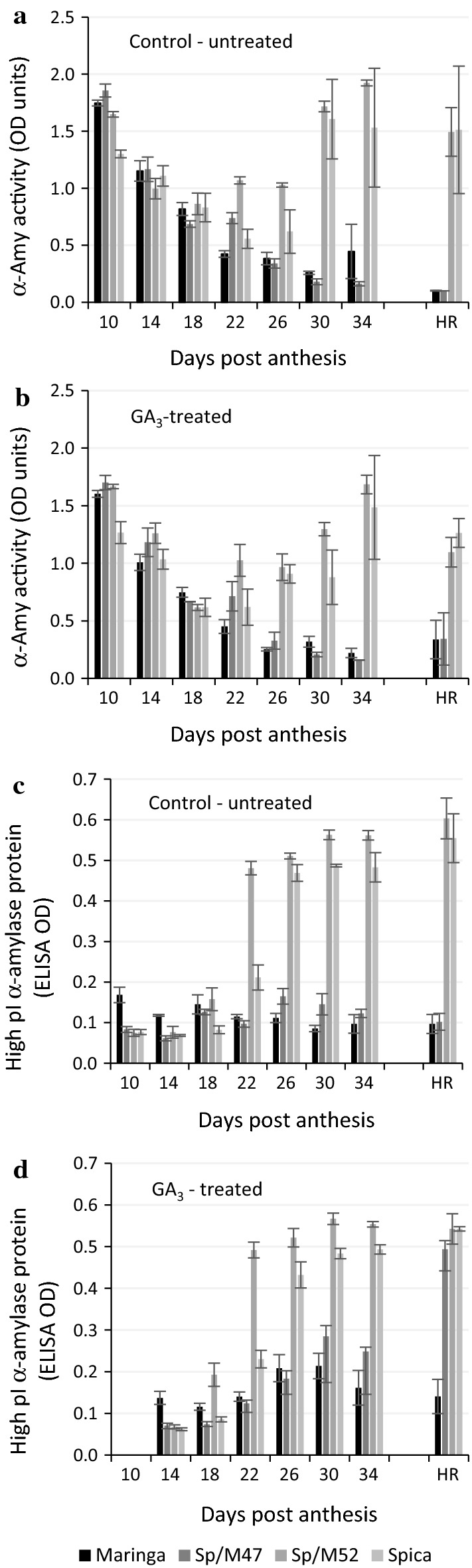
Total α-amylase activity and high pI α-amylase protein content in developing and harvest-ripe (HR) grain of control and GA_3_-treated plants of Maringa (LMA-resistant), Spica (LMA-susceptible), Sp/M47 (LMA-resistant) and Sp/M52 (LMA-susceptible). **a** Total α-amylase activity in grain from untreated plants. **b** Total α-amylase activity in grain from plants treated with GA_3_ at 10 days post anthesis. **c** High pI α-amylase protein content in grain of untreated plants. **d** High pI α-amylase protein content in grain from plants treated with GA_3_ at 10 days post anthesis. The experiment was conducted in triplicate and the error bars are standard errors

In a separate experiment, Maringa, Spica, Sp/M47 and Sp/M52 plants were sprayed with GA_3_ solution at 10 DPA with a follow-up spray at 15 DPA. Spikes, triplicate samples of 3 spikes, were harvested at 4-day intervals from 10 to 34 DPA and at harvest-ripeness and the grain analysed for GAs (22 DPA only in order to confirm the presence of GA_3_), total α-amylase activity (Megazyme Amylazyme™), high pI α-amylase protein (ELISA) and moisture content. The results were compared with grain from non-treated control plants. GA_3_ concentrations ranging from 4 to 25 µM were detected in grain samples harvested at 22 DPA. The time course of change in grain moisture content was not significantly different between varieties or treatments. Total α-amylase activity was high in all varieties at 10 DPA, had decreased by 18 DPA but then increased in Spica and Sp/M52 from 22 to 34 DPA and remained high in these lines at harvest-ripeness (Fig. 1a, b). Activity was lower in the GA_3_-treated samples of Spica and Sp/M52 compared with controls (Fig. 1a, b). The reasons for this reduction are not clear and appear to be inconsistent with the similar activity profiles for GA_3_-treated and controls up until 34 DPA. By contrast, total α-amylase activity continued to decrease in the LMA-resistant lines, Maringa and Sp/M47, although there was a small but not statistically significant increase in the GA_3_-treated samples at harvest-ripeness (Fig. 1b). By comparison, high pI α-amylase protein content was not significantly different from background (no colour-change in ELISA) until 22 DPA when a significant increase in Spica and Sp/M52 was recorded (Fig. 1c, d). Between 22 DPA and harvest-ripeness, high pI α-amylase content remained high and there was no significant difference (*P* = 0.05) between non-treated and GA_3_-treated Spica and Sp/M52. Between 26 and 34 DPA there appeared to be a slight, but statistically non-significant, increase in high pI α-amylase protein content in GA_3_-treated Maringa and Sp/M47 compared with untreated controls (Fig. 1d). There was a significantly higher high pI α-amylase protein content in the harvest-ripe samples of Sp/M47 but not Maringa (Fig. 1d), an observation that did not appear to be consistent with the α-amylase activity data for Sp/M47.

In a follow-up experiment, GA_3_ or ABA was injected into the peduncles of Sp/M52 and Sp/M47 plants. GA_3_ was subsequently detected in grains sampled at 23 and 29 DPA at concentrations ranging from 16 to 38 ng gDW^−1^. ABA concentrations in treated samples of Sp/M52 were 252 and 240 ng gDW^−1^ at 23 and 29 DPA respectively compared with 91 and 103 ng gDW^−1^ for the non-treated controls. Despite the presence of GA_3_ or the increase in ABA concentration, there were no significant differences in high pI α-amylase protein (ELISA OD) for grain at harvest- ripeness between GA_3_-treated, ABA-treated, or untreated controls. Similarly, treatment of Sp/M47 plants with GA_3_ resulted in a GA_3_ concentration in the grain of 16 ng gDW^−1^ at 23 DPA but in this experiment there was no detectable increase in ELISA OD for grain at harvest-ripeness relative to the background level of the non-treated control.

Pairs of lines with all possible combinations of Spica or Maringa alleles at the 7B, 3A and 2D QTL previously shown to be associated with variation in LMA were selected from a Spica (LMA-susceptible)/Maringa (LMA-resistant) doubled haploid population. GA_19_ concentrations measured at 26 DPA in lines carrying the Spica allele on chromosome 7B were similar to Spica parent irrespective of the allele at the other loci and more than ten-fold higher than Maringa or lines that only carried Spica alleles on chromosomes 3A or 2D (Table 2). Concentrations of GA_44_ (Supplementary Table S3) were more than twofold lower than GA_19_ but otherwise, the changes during grain development over time were similar. The lines with Maringa alleles at all three loci were similar to the Maringa parent. High pI α-amylase protein contents in harvest-ripe grain of Spica and lines that carried Spica alleles at the 7B locus were similar (Table 2). However, despite the very low GA_19_ concentrations, high pI α-amylase ELISA OD for lines with Spica alleles at only 3A or 2D, whilst lower than 7B lines, were higher than the background OD typical of Maringa and lines with Maringa alleles at all three loci.

**Table 2:** Grain gibberellin, GA_19_, concentration at 26 DPA and high pI α-amylase protein content (ELISA OD) in harvest-ripe (HR) grain of lines selected from a Spica (LMA-susceptible)/Maringa (LMA-resistant) doubled haploid population

Line ID	Allele	GA_19_ concentration ng gDW^−1^	High pI α-amylase at HR ELISA OD
Spica	**S7B, S3A, S2D**	73	**0.57 ± 0.02**
Maringa	M7B, M3A, M2D	4	0.10 ± 0.01
DM07.03.33	**S7B, S3A, S2D**	56	**0.69 ± 0.06**
DM07.03.120	**S7B, S3A, S2D**	93	**0.57 ± 0.13**
DM07.03.121	**S7B**, M3A, M2D	50	**0.52 ± 0.06**
DM07.03.192	**S7B**, M3A, M2D	44	**0.78 ± 0.04**
DM07.03.32	M7B, **S3A**, M2D	5	**0.45 ± 0.07**
DM07.03.42	M7B, **S3A**, M2D	4	**0.29 ± 0.17**
DM07.03.113	M7B, M3A, **S2D**	4	**0.38 ± 0.07**
DM07.03.128	M7B, M3A, **S2D**	4	**0.29 ± 0.07**
DM07.03.106	M7B, M3A, M2D	4	0.10 ± 0.03
DM07.03.154	M7B, M3A, M2D	4	0.09 ± 0.03

Pairs of lines were selected with Spica alleles (**S7B, S3A, S2D**) at LMA QTL on chromosomes 7B, 3A and 2D and compared with parental controls and a pair of lines with Maringa alleles (M7B, M3A, M2D) at these loci. Gibberellin concentrations were unreplicated whilst ELISA OD was determined on triplicate samples of harvest-ripe grain. DW = grain dry weight, standard errors are shown after the ± sign, HR = harvest-ripeness (grain moisture ≤ 12% fresh weight)

High pI α-amylase-specific ELISA values shown in bold were associated with a strong colour change in the assay and indicate that α-amylase protein was present. Values in plain font were not associated with a colour change and indicate that α-amylase protein was absent or below the level of detection

### Gibberellin concentration and high pI α-amylase protein content in reciprocal F_1_ grains

Duplicate samples, each with 2 or 3 spikes and a minimum of 25 F_1_ grains were harvested at 20, 25 and 30 DPA from parents, Spica (LMA-susceptible) and Maringa (LMA-resistant) and reciprocal F_1_s. Reciprocal hybrid F_1_ grains accumulated concentrations of GA_19_ and GA_44_ (data for GA_44_ in Supplementary Table S4) similar to the LMA parent Spica (Table 3). No gibberellins were detected in the LMA-resistant parent Maringa. In this case, despite the presence of high concentrations of GA_19_, the F_1_ grains had a non-LMA phenotype at harvest-ripeness with high pI α-amylase ELISA OD similar to Maringa.

**Table 3:** Gibberellin, GA_19_, concentration at 20, 25 and 30 DPA and high pI α-amylase protein content in harvest-ripe (HR) grain of parental varieties and reciprocal F_1_ hybrid grains

Line ID	DPA	GA_19_ concentration ng gDW^−1^	High pI α-amylase ELISA OD at HR
Spica	20	58 ± 10	
Spica	25	122 ± 5	
Spica	30	155 ± 14	
Spica	HR		**0.55 ± 0.02**
Maringa	20	nd	
Maringa	25	nd	
Maringa	30	nd	
Maringa	HR		0.09 ± 0.004
Spica/Maringa F_1_	20	80 ± 4	
Spica/Maringa F_1_	25	83 ± 23	
Spica/Maringa F_1_	30	116 ± 40	
Spica/Maringa F_1_	HR		0.075 ± 0.005
Maringa/Spica F_1_	20	45 ± 3	
Maringa/Spica F_1_	25	79 ± 6	
Maringa/Spica F_1_	30	117 ± 21	
Maringa/Spica F_1_	HR		0.10 ± 0.01

The first named parent of the hybrids is the female. Duplicate samples, each consisting of 2–3 spikes and a minimum of 25 F_1_ grains, were harvested at each sampling time. DW = grain dry weight, standard errors are shown after the ± sign

### Effect of a cool temperature shock on LMA phenotype and gibberellin concentration in semi-dwarf wheat varieties

The aim of these experiments was to determine whether the cool temperature shock required for an LMA phenotype to be expressed in some semi-dwarf genotypes when grown under warm conditions resulted in a change in gibberellin concentration. Two semi-dwarf lines, Hartog (LMA-resistant) and RAC655 (LMA-susceptible), both with *Rht-D1b*, were selected for this experiment based on consistent LMA phenotype over years (Derkx and Mares 2020; Derkx et al. 2021). Hartog carries haplotype H, the same as Spica, whilst RAC655 carries haplotype F of the *ent*-copalyl diphosphate synthase gene on chromosome 7B that has been linked to variation in LMA phenotype (Derkx et al. 2021). These haplotypes were distinct from the haplotypes A, B and C that were associated with LMA resistance, and both H and L groups contained a large number of varieties with LMA phenotype ranging from resistant to very susceptible (Derkx et al. 2021).

Triplicate samples of tillers, each replicate consisting of 3–5 tillers, harvested from plants of semi-dwarf varieties Hartog (LMA-resistant) and RAC655 (LMA-susceptible) were treated with a cool shock for 3, 5 or 7 days from 25 DPA (experiment 1) or 30 DPA (experiment 2). The experiments targeted two stages of the period of sensitivity to cool shock (Derkx and Mares 2020). Triplicate samples of control tillers were transferred to a warm environment for 3, 5 or 7 days. Grain from treated samples was frozen in liquid nitrogen and freeze-dried. Low concentrations of GA_8_ and much higher concentrations of both GA_19_ and GA_44_ were detected in all samples (Table 4). Overall, the GA concentration profiles were similar for the two varieties in experiment 1. By comparison, in experiment 2 the concentrations of GA_19_ and GA_44_ were not significantly different at 30 DPA but thereafter the concentrations of both declined more rapidly in Hartog than RAC655. There was a trend towards higher levels of GA_19_ and GA_44_ in the cool treated samples compared with controls. It should be noted that this difference may be due to the effect of the cool temperature in reducing the rate of grain development. Despite the similar GA profiles, high pI α-amylase protein ELISA OD remained at background levels for the LMA-resistant variety Hartog, both control and cool-treated, and the RAC655 control. An increase in ELISA OD was only observed with increasing treatment time for cool-treated RAC655 (LMA-susceptible). High pI α-amylase protein was also not detected in samples of Hartog or RAC655 harvested from untreated plants that remained in the glasshouse until harvest-ripeness (HR).

**Table 4:** Effect of a cool temperature shock on gibberellin concentration and LMA phenotype of semi-dwarf lines RAC655 (LMA) and Hartog (non-LMA)

Line ID	DPA/treatment	GA_8_ ng gDW^−1^	GA_19_ ng gDW^−1^	GA_44_ ng gDW^−1^	High pI α-amylase ELISA OD
Experiment 1					
Hartog	25	10	148	249	0.061 ± 0.002
Hartog	25 + 3d cool	12	151	212	0.073 ± 0.004
Hartog	25 + 3d warm	9	136	249	0.074 ± 0.004
Hartog	25 + 5d cool	No data	No data	No data	0.069 ± 0.007
Hartog	25 + 5d warm	No data	No data	No data	0.067 ± 0.005
Hartog	25 + 7d cool	7	118	151	0.12 ± 0.018
Hartog	25 + 7d warm	9	78	112	0.070 ± 0.004
Hartog	HR	No data	No data	No data	0.071 ± 0.007
RAC655	25	4	165	88	0.099 ± 0.006
RAC655	25 + 3d cool	8	155	49	**0.20 ± 0.018**
RAC655	25 + 3d warm	10	134	130	0.10 ± 0.012
RAC655	25 + 5d cool	No data	No data	No data	**0.40 ± 0.034**
RAC655	25 + 5d warm	No data	No data	No data	0.16 ± 0.016
RAC655	25 + 7d cool	7	134	52	**0.56 ± 0.057**
RAC655	25 + 7d warm	7	69	112	0.19 ± 0.026
RAC655	HR	No data	No data	No data	0.14 ± 0.026
Experiment 2					
Hartog	30	9 ± 1	41 ± 4	113 ± 8	0.073 ± 0.001
Hartog	30 + 3d cool	5 ± 1	35 ± 3	54 ± 8	0.084 ± 0.007
Hartog	30 + 3d warm	8*	16 ± 2	25 ± 6	0.072 ± 0.001
Hartog	30 + 5d cool	nd	23 ± 6	43 ± 17	0.089 ± 0.004
Hartog	30 + 5d warm	nd	13 ± 7	18 ± 9	0.068 ± 0.001
Hartog	30 + 7d cool	nd	17 ± 1	14 ± 1	0.074 ± 0.002
Hartog	30 + 7d warm	5*	6 ± 1	10 ± 2	0.071 ± 0.005
Hartog	HR	No data	No data	No data	0.066 ± 0.006
RAC655	30	7 ± 2	53 ± 4	93 ± 3	0.096 ± 0.004
RAC655	30 + 3d cool	9 ± 1	76 ± 3	94 ± 14	**0.27 ± 0.045**
RAC655	30 + 3d warm	11 ± 1	59 ± 1	79 ± 9	0.17 ± 0.027
RAC655	30 + 5d cool	9 ± 2	69 ± 10	57 ± 10	**0.25 ± 0.013**
RAC655	30 + 5d warm	9 ± 3	48 ± 8	49 ± 6	0.15 ± 0.023
RAC655	30 + 7d cool	7 ± 1	51 ± 6	31 ± 3	**0.47 ± 0.070**
RAC655	30 + 7d warm	4*	21 ± 5	20 ± 4	0.113 ± 0.006
RAC655	HR	No data	No data	No data	0.089 ± 0.007

Experiment 1: Tillers detached from glasshouse plants at 25 DPA and subjected to a cool temperature shock of 3, 5 or 7 days duration. Controls were maintained under warm conditions for the same periods. Experiment 2: As for experiment 1 except that tillers were taken from plants at 30 DPA

*HR*  grain samples collected from untreated plants at harvest-ripeness, DW = dry weight, standard errors are shown after the ± sign

*Indicates the GA_8_ was detected in one of the 3 replicates analysed

High pI α-amylase-specific ELISA values shown in bold were associated with a strong colour change in the assay and indicate that α-amylase protein was present. Values in plain font were not associated with a colour change and indicate that α-amylase protein was absent or below the level of detection

### Changes in the response of de-embryonated grains to exogenous gibberellin, GA_3_, during grain development and ripening

**Fig. 2:**
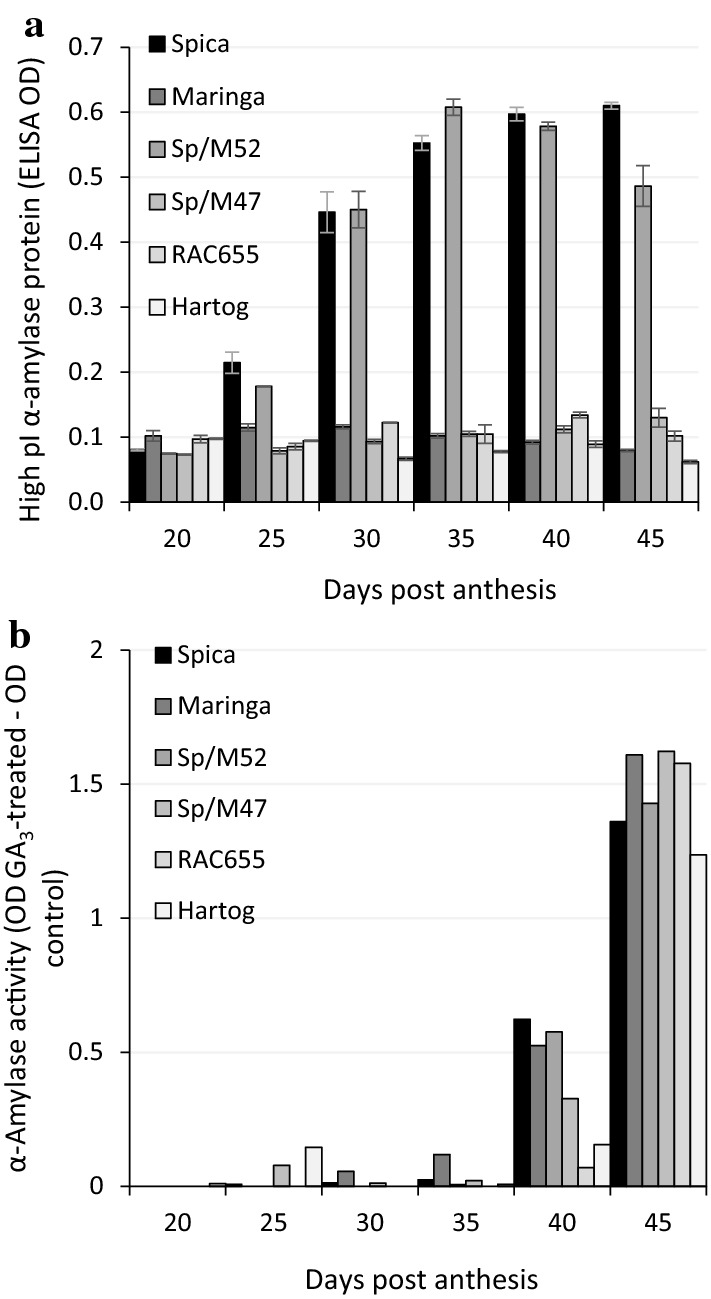
High pI α-amylase protein content and total α-amylase activity in developing grain of control and GA_3_-treated distal halves of grains. **a** High pI α-amylase protein content in distal halves of grains sampled at intervals after anthesis and determined as high pI α-amylase ELISA OD. **b** Total α-amylase activity in distal halves of grains sampled at intervals after anthesis and incubated with exogenous GA_3_ or water (control). In order to avoid confounding effects due to synthesis of high pI α-amylase in the absence of exogenous GA_3_ in the tall LMA-susceptible lines, Spica and Sp/M52, activity was calculated as OD GA_3_ treated – OD water control. The experiment was conducted in triplicate

Grains were harvested at 5-day intervals, commencing at 20 DPA, the proximal (embryo) end excised and the de-embryonated sections incubated in petri dishes on filter paper moistened with water, control, or 20 µM GA_3_. High pI α-amylase protein content in the absence of exogenous GA_3_, determined on the control samples using ELISA, increased in both Spica and Sp/M52 starting at 25 DPA (Fig. 2a) but not Maringa, Sp/M47, Hartog or RAC655. To determine the capacity to respond to exogenous GA when some varieties were already producing α-amylase in the absence of exogenous GA, total α-amylase activity was determined on control and GA-treated samples and the mean OD for water controls was subtracted from the mean OD of GA_3_-treated samples. For the tall varieties, Spica, Maringa, Sp/M52 and Sp/M47, the capacity to respond to exogenous GA_3_ was not achieved until 40–45 DPA (Fig. 2b). For the semi-dwarf varieties, Hartog and RAC655, the difference was quite small at 40 DPA but similar to tall varieties by 45 DPA. Interestingly, the time course of change in response to GA was similar for all varieties examined irrespective of their LMA phenotype and the capacity to respond to exogenous GA_3_ was not acquired until some 15 days later than the synthesis of α-amylase in Spica and Sp/M52 in the absence of exogenous GA_3_.

### Determination of GA_55_

GA_55_ was detected in the tall LMA-susceptible lines but was absent in grain from the tall LMA-resistant lines and Spica plants that had been treated with paclobutrazol (Table 5). This gibberellin was also detected in both semi-dwarf lines, Hartog (LMA-resistant) and RAC655 (LMA-susceptible), irrespective of the warm or cool temperature treatments although there was a trend towards higher concentrations in the LMA-susceptible line RAC655. High pI α-amylase protein ELISA OD readings greater than the background were observed for Spica, Sp/M52, Sp/M52 + paclobutrazol, and the RAC655 cool temperature treated samples.

**Table 5:** GA_55_ concentration and high pI α-amylase protein content in developing grain of tall lines Spica (LMA-susceptible), Sp/M52 (LMA-susceptible), Maringa (LMA-resistant) and Sp/M47 (LMA-resistant) determined at intervals during early grain development (experiment 1), and in grain of semi-dwarf lines RAC655 (LMA) and Hartog (non-LMA) with and without cool shock

Line	DPA	GA_55_ ng gDW^−1^	High pI α-amylase protein ELISA OD
Experiment 1			
Spica	14	29 ± 2	0.07 ± 0.01
Spica	18	44 ± 2	0.08 ± 0.01
Spica	22	59 ± 2	**0.21 ± 0.03**
Spica	26	44 ± 2	**0.47 ± 0.04**
Maringa	14	nd	0.12 ± 0.00
Maringa	18	nd	0.14 ± 0.02
Maringa	22	nd	0.11 ± 0.01
Maringa	26	nd	0.11 ± 0.01
Sp/M52	14	nd	0.08 ± 0.02
Sp/M52	18	37 ± 2	0.16 ± 0.01
Sp/M52	22	38 ± 1	**0.48 ± 0.01**
Sp/M52	26	45 ± 4	**0.51 ± 0.01**
Sp/M47	14	nd	0.06 ± 0.01
Sp/M47	18	nd	0.13 ± 0.01
Sp/M47	22	nd	0.10 ± 0.01
Sp/M47	26	nd	0.16 ± 0.02
Spica + paclo	18	nd	0.18 ± 0.01
Spica + paclo	26	nd	**0.51 ± 0.04**
Maringa + paclo	18	nd	0.11 ± 0.01
Maringa + paclo	26	nd	0.10 ± 0.01
Experiment 2			
RAC655 + warm	14	36 ± 1	0.14 ± 0.01
RAC655 + warm	18	48 ± 2	0.15 ± 0.01
RAC655 + warm	22	48 ± 2	0.16 ± 0.03
RAC655 + warm	26	51 ± 3	0.16 ± 0.03
RAC655 + cool	14	49 ± 3	0.26 ± 0.04
RAC655 + cool	18	66 ± 2	**0.29 ± 0.05**
RAC655 + cool	22	61 ± 6	**0.47 ± 0.03**
RAC655 + cool	26	30 ± 4	**0.55 ± 0.02**
Hartog + warm	14	22 ± 2	0.08 ± 0.01
Hartog + warm	18	31 ± 1	0.09 ± 0.01
Hartog + warm	22	26 ± 1	0.08 ± 0.01
Hartog + warm	26	21 ± 3	0.09 ± 0.01
Hartog + cool	14	33 ± 6	0.09 ± 0.005
Hartog + cool	18	23 ± 1	0.12 ± 0.02
Hartog + cool	22	23 ± 2	0.18 ± 0.02
Hartog + cool	26	nd	0.13 ± 0.03

*nd*  GA_55_ not detected

High pI α-amylase-specific ELISA values shown in bold were associated with a strong colour change in the assay and indicate that α-amylase protein was present. Values in plain font were not associated with a colour change and indicate that α-amylase protein was absent or below the level of detection

### Transcript abundance of genes involved in GA biosynthesis

**Fig. 3:**
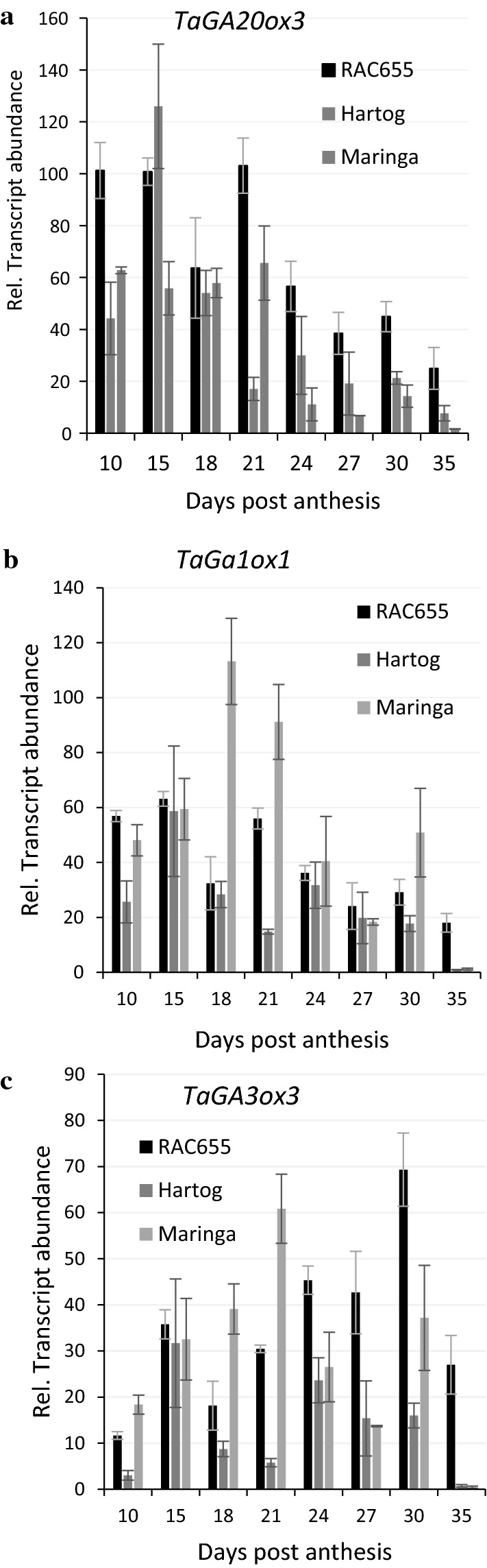
The relative fold difference of **a** GA 20-oxidase (*TaGA20ox3*), **b** GA 1-oxidase, *TaGA1ox1*, and **c** GA 3-oxidase, *TaGA3ox3* transcripts in de-embryonated developing grains of three wheat lines, RAC655, Hartog and Maringa, sampled in triplicate, 20 seeds per replicate, at intervals from 10 to 30 DPA. Fold differences in gene transcripts were calculated as relative fold increase using the PFAFFL method (Pfaffl 2001) and control transcript Actin. Standard errors shown after the ± signs were calculated in Excel

Transcript abundance of GA 1-oxidase and GA 3-oxidase genes reported by Pearce et al. (2015) to be involved in the synthesis of the 1-β-hydroxy gibberellins such as GA_55_ and GA_54_ found in developing wheat grains (Gaskin et al. 1980; Lenton and Gale 1987), as well as GA 20-oxidase were determined at intervals during grain development. Transcripts of each these genes were detected from an early stage in both RAC655 and Hartog (Fig. 3) which synthesised GA_55_. However, similar levels of gene transcripts were also detected in developing grains of the LMA-resistant line Maringa where neither GA biosynthesis intermediates nor GA_55_ could be detected.

## Discussion

Is the mechanism involved in α-amylase synthesis during grain development (LMA) similar to that in aleurone of ripe grain during germination or following treatment with exogenous gibberellin? In other words, is LMA dependent on the synthesis of gibberellins and a functional GA transduction pathway that includes in sequence the deactivation of the negative regulator, DELLA, the activation of GAMyb and the subsequent stimulation of transcription of the *TaAmy1* genes?

As noted above, a role for DELLA and hence GA in LMA could be inferred from the reduction of LMA expression in semi-dwarf, GA-insensitive wheat varieties when compared with GA sensitive tall varieties, or lines that have a semi-dwarf habit conferred by mechanisms other than GA insensitivity for example *Rht8* in the variety ChuanMai18 (Mrva et al. 2008). GA insensitive semi-dwarf wheat lines carry a mutation of a DELLA gene on either chromosome 4B (*RhtB-1b*) or chromosome 4D (*RhtD-1b*), that impact the GA signalling pathway. The interpretation of data for GA-insensitive semi-dwarfs requires some caution since the mutations are not fully GA-insensitive and the response to GA will reflect the sum of effects of one DELLA mutant in the presence of a GA-sensitive DELLA gene. In relation to LMA, this inhibitory action is less pronounced when plants are grown at cooler temperatures (Derkx and Mares 2020) but provides effective resistance when plants are grown at warmer temperatures. This resistance is overcome if plants are subjected to a cool temperature shock during grain development (Mrva and Mares 2001a; Mrva et al. 2006; Farrell and Kettlewell 2008; Derkx and Mares 2020) although the mechanism involved remains unclear. The cool temperature shock did not have a significant impact on GA_19_ concentration but may have affected GA sensitivity. The possibility that the cool shock had an impact on α-amylase protein turnover was also considered but not specifically addressed in this study. In all the experiments on developing wheat grains conducted, high pI α-amylase protein reached a peak content that appeared to be retained until harvest-ripeness. Exposure of LMA susceptible semi-dwarf genotypes that had been grown under warm temperatures to a cool shock during grain development, resulted in synthesis of high pI α-amylase protein similar to non-treated tall genotypes. In comparison with developing grain, synthesis of α-amylase is greatly reduced in germinating grain of *Rht-B1c* extreme dwarfs at 25 °C but not at 15 °C (Lenton and Appleford 1993). Singh and Paleg (1984, 1985) reported that low temperatures increased the GA_3_ sensitivity, measured as α-amylase production, in isolated aleurone tissue of varieties with *Rht-B1b*, *Rht-D1b* or *Rht-B1c*. The inhibitory effect of *Rht-B1b* at warm temperatures was also overcome in the presence of the 1B/1R wheat/rye translocation in the variety Seri82 (Mrva et al. 2008).

Developing grains of some LMA lines accumulate high concentrations of GA biosynthesis intermediates as well as transcripts of *ent*-CPS, the first committed step in the biosynthesis of gibberellins (Barrero et al. 2013; Derkx et al. 2021). However, as pointed out by Derkx et al. (2021) these observations do not provide unequivocal proof that GA is involved in LMA.

The results presented in this study confirmed that gibberellin biosynthesis intermediates as well as the bioactive GA_55_ do accumulate in grain of varieties that develop an LMA phenotype and that the genes likely to be involved in synthesis of 1-β-hydroxy GAs such as GA_55_, as well as *TaGA20ox3* (Pearce et al. 2015) were transcribed from an early stage in grain development even in the LMA-resistant variety, Maringa. But clearly, there are instances where this apparent linkage is decoupled. Following treatment of wheat spikes with paclobutrazol shortly after flowering, gibberellins failed to accumulate but an LMA phenotype was still observed. Earlier publications (Gaskin et al. 1980; Lenton et al. 1994; Appleford and Lenton 1997) provided strong evidence that substantial concentrations of GA intermediates such as GA_19_ accumulated in both developing and germinating wheat grains. In developing grain there was some conversion of these intermediates into high concentrations of novel 1-β-hydroxy gibberellins such as GA_54_ and GA_55_ (Gaskin et al. 1980; Lenton and Gale 1987) and GA_55_ (this study) that disappeared during ripening and were not detected in mature grain (Lenton et al. 1994). GA_1_, GA_4_ or GA_3_ were not detected in the developing grains however they may have been present at levels below that required for detection. GA_1_ and GA_3_, but no 1-β-hydroxy GAs, were detected in germinating grains (Lenton et al. 1994). These observations suggest that GA synthesis is different in developing compared with germinating wheat grains. In contrast to these observations, Kondhare et al. (2014a) detected both GA_1_ and GA_3_ in developing grains of Rialto at 580° days after anthesis. The reason for these contrasting observations for developing grains is unclear but may be related to the different genetics used in the respective studies and requires further investigation.

Treatment of spikes with paclobutrazol shortly after anthesis resulted in a failure to synthesise GA intermediates, GA_19_ and GA_44_, and the bioactive gibberellin GA_55_ to accumulate during grain development in the LMA susceptible line Sp/M52. Despite this absence of gibberellins, there was a high accumulation of high pI α-amylase protein that was not significantly different from the non-treated control.

By comparison, when prohexadione-Ca was applied there was a very substantial build-up of intermediates such as GA_19_ and GA_44,_greater than the untreated control, that suggested the pathway was blocked at the stage of conversion of biologically inactive to biologically active gibberellins. Whilst information on the pharmacology of this inhibitor is limited, Nakayama et al. (1992) concluded that, whilst it inhibited a series of steps from GA_20_ to GA_1_ catalysed by GA20 oxidase, the primary mode of action of prohexadione-Ca was the final 3β-hydroxylation step in the conversion of GA_20_ to GA_1_. Yet again the LMA phenotype was strongly expressed. As a consequence, it appeared that substantial perturbations of the concentrations of GA biosynthesis intermediates were not reflected in a significant change in high pI α-amylase protein content.

An important caveat here is that these inhibitors may be impacting other hormone biosynthesis pathways or may not be reaching the target tissues within the grain. The latter appears unlikely given the dramatic changes in GAs that were observed. Similarly, Mrva et al. (2006) reported that the LMA response was associated with changes in a relatively small number of isolated cells distributed within the aleurone tissue. It is possible that these cells synthesise bioactive gibberellins but these are not detected when hormone measurements are from entire grains. This issue could be addressed in future using GA FRET biosensor technology (Rizza et al. 2021).

When spikes of LMA-susceptible lines, Spica and Sp/M52, or LMA-resistant lines, Maringa and Sp/M47, were treated with exogenous GA_3_, there was no significant change in the LMA phenotype (ELISA OD) for Spica or Sp/M52. Results for the LMA-resistant lines were inconsistent. In one experiment there was no evidence of synthesis of high pI α-amylase in the non-LMA line Sp/M47 despite the presence of exogenous-derived GA_3_ at the time when LMA-susceptible lines accumulated α-amylase protein. In a second experiment, there was no increase in α-amylase activity in the LMA-resistant lines Sp/M47 and Maringa, but an apparent accumulation of α-amylase protein late in grain development, particularly evident in the harvest-ripe grain, in Sp/M47 but not Maringa. ABA has been shown to reduce the synthesis of α-amylase during germination. In this study, application of exogenous ABA shortly after anthesis was reflected in an increased ABA concentration in the grains but there was no significant reduction in the accumulation of α-amylase protein in the LMA-susceptible line Sp/M52.

The results obtained following treatment of plants with GA_3_ or ABA in this study are not consistent with Kondhare et al. (2012, 2014b) who reported an increase and a reduction in total α-amylase activity, respectively. As noted previously, this lack of consistency between the studies may be related to the different genetic material used. Kondhare et al. (2012, 2013, 2014a) used the LMA-susceptible variety Rialto which carries *Rht-D1b* as well as the 1B/1R wheat/rye translocation (Farrell et al., 2013). Farrell et al. 2013 reported that the 1B/1R translocation increased the expression of LMA and acted independently of the *Rht-D1* alleles. Mrva and Mares (2008) had earlier reported that the 1B/1R translocation was associated with increased LMA expression in Seri82 compared with a Seri82 isoline that lacked the translocation. None of the wheat varieties used in the current study contain the 1B/1R translocation. Further work is required to resolve the cause of the differences.

Kondhare et al. (2014b) applied tritiated ABA or GA_3_ to confirm that the hormones were taken up by the grain. Since the application methods used were similar to those used in the current study, the inconsistency is unlikely to be due to significant differences in hormone uptake. Differences between the studies include: (i) the timing of hormone application, 10 DPA in the current study versus 26 DPA (Kondhare et al. 2012); (ii) α-amylase determination at intervals throughout grain development until harvest-ripeness in the current study versus measurement at maturity only by Kondhare et al. (2012); and (iii) determination of both total α-amylase activity and high pI α-amylase protein in the current study versus total α-amylase activity only by Kondhare et al. (2012) and the possibility that GA might regulate activity rather than protein accumulation. Comparison of the two phenotyping methodologies is difficult for developing grains due to the presence of low pI α-amylase particularly in the early phases of grain development which is not detected by the high pI-specific α-amylase ELISA. During the later stages of grain development low pI α-amylase disappears and the confounding effect is much less of an issue. For the experiment where both activity and protein content were determined in parallel, the simple correlation coefficient (*R*^2^) was 0.845. Furthermore, calculation using the activity and protein content data presented by Derkx and Mares (2020) for field expression of LMA, gave a correlation coefficient (*R*^2^) of 0.87. The second of these differences in methodology is perhaps the most significant, since the regular sampling throughout grain development allowed an accurate time of changes to be determined and any effects of the exogenous GA_3_ application to be aligned temporally with the naturally occurring synthesis of α-amylase in an LMA-susceptible genotype. In the current study, there was evidence that α-amylase protein had only increased late in grain ripening in Sp/M47 consistent with the in vitro experiment where de-embryonated grains did not respond to exogenous GA_3_ until around 40 DPA when the grains near maximum dry weight.

Throughout this study, the term high pI α-amylase has been used to describe the α-amylase isoforms present in the endosperm of LMA-affected grain. Based on the work by Gale et al. (1983) and Mares and Gale (1990) these isoforms appeared to be products of the *TaAmy1* genes. *TaAmy3* genes associated with very high pI α-amylases were not expressed in LMA (Barrero et al. 2013). During the latter stages of this study, Meog et al. (2017) reported the identification of a fourth group of α-amylase genes, *TaAmy4*, located on the group 5 chromosomes. No proteins were isolated, however the isolectric points of the proteins were predicted to be between pH 6.0 and 6.27 and if present would likely run between low pI Amy2 and high pI Amy1 on IEF gels. The *TaAmy4* genes were expressed at a low level in LMA (Barrero et al. 2013) and both Meog et al. (2017) and Newberry et al. (2018) suggested that *TaAmy4* could contribute to the LMA phenotype. The ELISA used in this study does not detect low pI α-amylase, Amy2, (Mares and Mrva, unpublished data) but it is not clear whether it detects Amy4 or Amy3 in addition to Amy 1. Whilst resolution of this issue is outside the scope of the current work, future work might be needed to define precisely the α-amylase proteins recognised by the ELISA.

Lines from a Spica (LMA)/Maringa (non-LMA) population that carried minor genetic loci for LMA from Spica but have the non-LMA allele from Maringa at the major 7B locus (Derkx et al. 2021) did not accumulate gibberellins but did show an LMA phenotype, albeit reduced in magnitude when compared with the LMA parent, Spica. In contrast, semi-dwarf, *Rht-D1b*, wheat lines Hartog and RAC655 both accumulated GA biosynthesis intermediates when grown in a warm, > 25 °C daily maximum temperature, environment in which both exhibited a non-LMA phenotype. An LMA phenotype was induced in RAC655 but not Hartog following a cool temperature shock and in RAC655 but not Hartog when these varieties were maintained at lower temperatures (Derkx and Mares 2020). Similarly, reciprocal F_1_ hybrid grains produced by crossing Spica with Maringa accumulated gibberellins but maintained a non-LMA phenotype. Finally, the results obtained in this study confirmed an earlier report (Armstrong et al. 1982) that wheat aleurone did not acquire the capacity to respond to exogenous GA_3_ until very late in grain ripening. This timing was 15 days after the synthesis of α-amylase and possibly more likely associated with the preparation of the grain for germination.

An additional confounding factor is the high concentrations of ABA in both LMA-susceptible and LMA-resistant varieties. ABA has been reported to inhibit GA transduction by inhibiting the transcription of GAMyb (Gomez-Cadenas et al. 2001) and also at the stage of α-amylase gene transcription (Nolan et al. 1987; Zwar and Hooley 1996). Application of ABA to spikes of Sp/M52 as part of the current study failed to reduce the synthesis of α-amylase. Thus, on the one hand, it is possible to have an LMA phenotype in the apparent absence of GA and with high concentrations of the GA-antagonist ABA. Conversely, the presence of GA does not guarantee expression of LMA.

This raises several questions including: what is the function of the novel GAs that are synthesized in developing grains, and how do DELLA mutants interfere with the expression of LMA at warmer temperatures? Radley (1976) reported that maximum grain volume is inversely related to temperature and is reached while the dry weight is still small. This stage is associated with a sharp increase in gibberellins leading Radley (1976) to suggest that GA was associated with cell expansion during early grain development. More recently, Wanchoo-Kohli (2017) showed that GA in developing endosperm is involved in regulating grain size and morphology and in addition showed that manipulation of GA content did not produce differences in α-amylase levels. The temporal overlap of GA synthesis and LMA in some lines may simply be a coincidence. Whilst the mechanism involved in the effects of DELLA mutants on LMA is unclear, it seems possible that the mechanism behind their effect on LMA phenotype involves a function of DELLA other than its well-established role in GA signal transduction. This is of course pure speculation. The decreased LMA susceptibility in *Rht-B1b* and *Rht-D1b* lines, particularly when plants are grown under warm temperatures, suggests that their impact on GA signalling remains the more likely cause. Clearly this is an area that requires further research.

Genetic evidence strongly supports the role of CPS in LMA (Derkx et al. 2021). This enzyme catalyses the first committed step in the synthesis of gibberellins from geranylgeranyl diphosphate. Paclobutrazol inhibits a later step in the pathway catalysed by *ent*-kaurine oxidase and in this study was shown to inhibit the synthesis of gibberellins without inhibiting the synthesis of α-amylase. This suggests that the product of CPS or the next enzyme in the pathway, *ent*-kaurine synthase, copalyl diphosphate or *ent*-kaurene respectively, might feed into an alternate pathway in developing grains that ultimately leads to the synthesis of α-amylase. Derkx et al. (2021) reported that some LMA-resistant varieties transcribed CPS and produced gibberellins and proposed that other genetic loci could reduce the impact of a functional CPS gene. Potentially such loci could interact with an alternate pathway to α-amylase synthesis if it exists. The CPS associated with LMA (Derkx et al. 2021) is similar in sequence to other CPS genes that are involved in gibberellin biosynthesis (Toyomasu et al. 2009; Wu et al. 2012). However, whilst the *ent*-copalyl diphosphate protein included the aspartate-rich DxDD and a histidine-asparagine (H-N) dyad catalytic motifs that are known features of CPS proteins involved in GA biosynthesis (Prisic et al. 2007; Lemke et al. 2019), a histidine that has been reported to act as a regulatory switch is replaced by arginine. Mann et al. (2010) concluded that this change would make the CPS insensitive to inhibition by Mg^2+^ and allow the rapid production of secondary metabolites.

## Conclusions

This work demonstrates that high pI α-amylase synthesis in the aleurone of developing wheat grains during LMA expression may be independent of gibberellins. The control of α-amylase synthesis may therefore be quite different to that reported for α-amylase synthesis in the aleurone of ripe grains during germination or following treatment of aleurone tissue with exogenous gibberellin. As a consequence, further work is required to identify the biochemical and molecular components of the pathway in developing grain that leads to the coordinated and specific synthesis of high pI α-amylase by the *α-Amy-1* genes located on the long arms of the group six chromosomes. Similarly, whilst the function of the particular *ent*-copalyl diphosphate synthase (CPS) gene linked with LMA remains unclear it appears to be separate from its role in gibberellin biosynthesis. Putative genetic loci located elsewhere on the genome that appear to modulate the LMA-specific function of this CPS gene are currently under investigation. Finally, further research is needed to understand how mutants of the negative regulator of gibberellin signalling, DELLA, in semi-dwarf and dwarf wheat varieties reduce LMA expression of LMA under warm but not cooler temperatures.

### *Author contribution statement*

DM and AD conceived and designed research. DM and AD conducted experiments to provide the samples for hormone and gene expression analyses. JC performed the gene transcript analysis, IZ performed the plant hormone analysis, KM was responsible for the GA-sensitivity experiment, RA synthesised the GA_55_ and carried out the studies on its activity. DM wrote the manuscript with input from the other authors. All authors read and approved the manuscript.

## Supplementary Information

Below is the link to the electronic supplementary material.

Supplementary file1 (DOCX 30 KB)

Supplementary file2 (XLSX 12 KB)

Supplementary file3 (XLSX 13 KB)

Supplementary file4 (XLSX 14 KB)

Supplementary file5 (PPTX 3264 KB)

Supplementary file6 (DOCX 210 KB)

Supplementary file7 (PPTX 42 KB)

## Data Availability

All data generated or analysed during this study are included in this published article (and its supplementary information files).

## Abbreviations

CPS: Copalyl diphosphate synthase
DPA: Days post-anthesis
ELISA: Enzyme-linked immunosorbent assay
GA: Gibberellin
LMA: Late-maturity α-amylase
pI: Isoelectric point
Rht: Reduced height
