# Supplementary material for: Gibberellins in developing wheat grains and their relationship to late maturity α-amylase (LMA)
Source: Planta. 2022 May 6;255(6):119. doi: 10.1007/s00425-022-03899-y (PMC9076747; doi:10.1007/s00425-022-03899-y)
Supplement: Supplementary file 1 — Supplementary file1 (DOCX 30 KB) [file 425_2022_3899_MOESM1_ESM.docx]

Supplementary Table S1. Confirmation of the structure of gibberellin GA_55_ using nuclear magnetic resonance (NMR). Assignments (Assign.) were established by proton (^1^H) and carbon (^13^C) spectra along with data from two dimensional ^1^H-^13^C heteronuclear multiple bond coherence spectroscopy (HMBC) and ^1^H-^1^H correlation spectroscopy (COSY). Proton-proton and carbon-carbon spin coupling constants (*J* values) for doublet (d) peaks are indicated and peaks with greater splitting are shown as multuplets (m). No triplet peaks were observed. The number of protons were calculated using proton peak areas.

| Assign.^a^ | ^13^C  (δ ppm) | ^1^H  (δ ppm) | No. protons | HMBC  correlations | COSY  correlations |
| --- | --- | --- | --- | --- | --- |
| 7 | 183.0 |  | 0 |  |  |
| 19 | 180.6 d  (*J* = 65.5Hz) |  | 0 |  |  |
| 16 | 158.5 |  | 0 |  |  |
| 17 | 108.0 | 4.982 | 2 | C13, C14, C16 | H14a, H14b |
|  |  | 4.811 |  | C12, C13, C14, C16 | H12, H14a, H14b, H15 |
| 10 | 98.6 |  | 0 |  |  |
| 13 | 79.5 |  | 0 |  |  |
| 3 | 71.5 | 3.578 | 1 | C1, C2, C5, C8, C18 | H1, H2, H5, H18 |
| 1 | 65.8 | 3.827 | 1 | C2, C3, C5, C10 | H2, H3 |
| 8 | 56.5 |  | 0 |  |  |
| 6 | 56.0 | 2.379 d  (*J* = 9.91Hz) | 1 | C1, C4, C5, C8, C14, C15, C18 | H5 |
| 4 | 50.1 |  | 0 |  |  |
| 9 | 48.4 | 2.05 | 1 | C1, C3, C4, C10, C11, C12, C14, C15 | H11, H12 |
| 5 | 48.0 | 3.29 d  (*J* = 9.99Hz) | 1 | C1, C3, C6, C7, C8, C18 | H6 |
| 15 | 46.5 | 1.72 d  (*J* = 10.8Hz) | 1 | C4, C6, C9, C12, C13, C14, C16 | H14, H15 |
|  |  | 1.58 d  (*J* = 10.7Hz) | 1 | C4, C6, C9, C12, C13, C14, C16 | H14, H15 |
| 14 | 44.3 | 2.17 m | 1 | C4, C9, C13, C16, C17 | H14, H15, H17a, H17b |
|  |  | 2.06 m | 1 | C4, C9, C13, C16, C17 | H14, H15, H17a, H17b |
| 12 | 39.6 | 1.77 m, 1.58 m | 2 | C9, C11, C16 | H9, H11 |
| 2 | 37.0 | 1.80 m | 2 | C1, C3, C8, C10 | H1, H3 |
| 11 | 18.0 | 1.59 m, 1.44 m | 2 | C4, C9, C10, C12, C13, C15 | H9, H12, H11 |
| 18 | 15.3 | 0.99 | 3 | C3, C5, C6, C7 | H2, H3, H5 |

^a^
