# Supplementary material for: Gibberellins in developing wheat grains and their relationship to late maturity α-amylase (LMA)
Source: Planta. 2022 May 6;255(6):119. doi: 10.1007/s00425-022-03899-y (PMC9076747; doi:10.1007/s00425-022-03899-y)
Supplement: Supplementary file 5 — Supplementary file5 (PPTX 3264 KB) [file 425_2022_3899_MOESM5_ESM.pptx]

## Slide 1
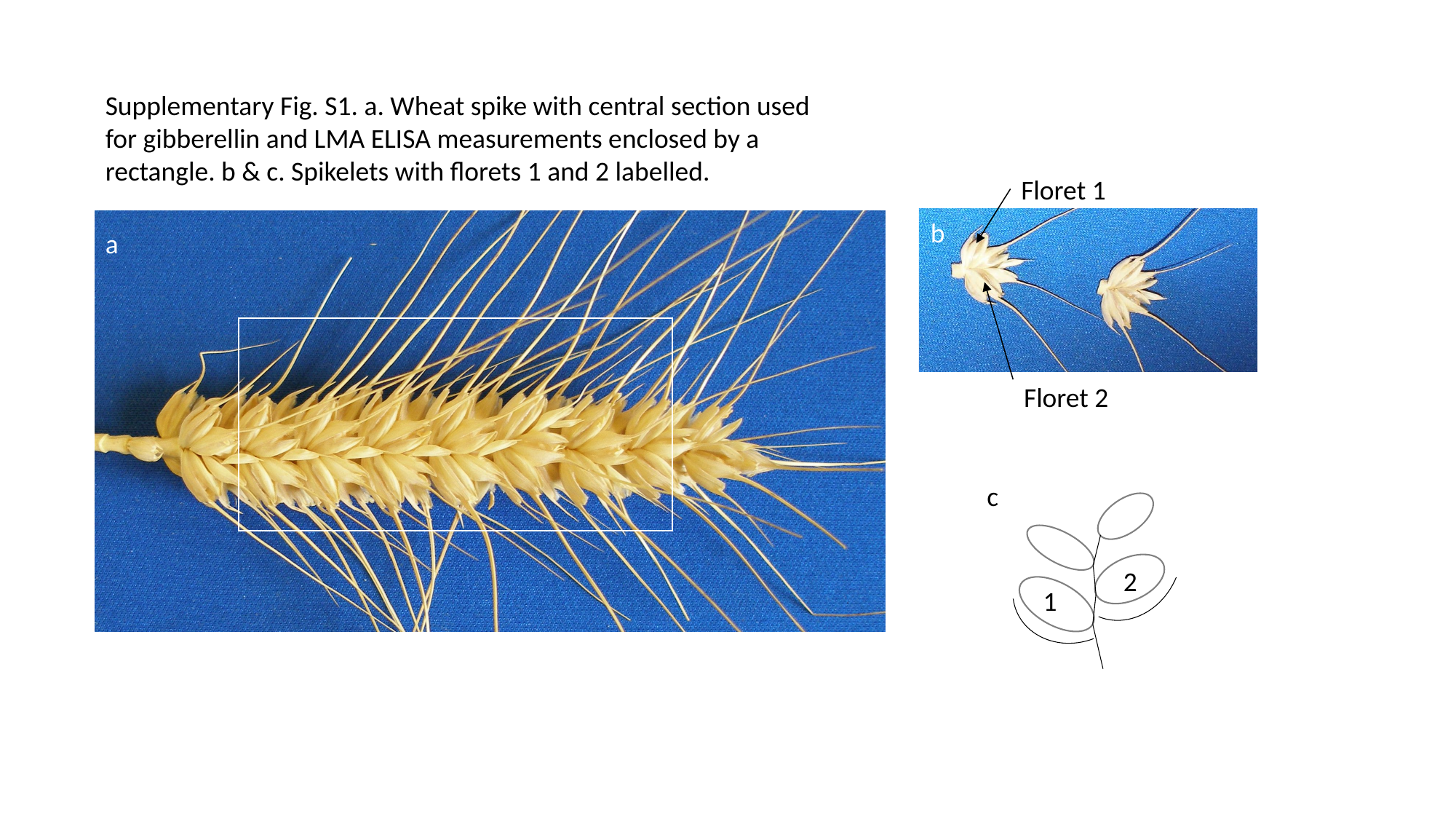

Supplementary Fig. S1. a. Wheat spike with central section used for gibberellin and LMA ELISA measurements enclosed by a rectangle. b & c. Spikelets with florets 1 and 2 labelled.
Floret 1
b
a
Floret 2
c
2
1
