# Supplementary material for: Gibberellins in developing wheat grains and their relationship to late maturity α-amylase (LMA)
Source: Planta. 2022 May 6;255(6):119. doi: 10.1007/s00425-022-03899-y (PMC9076747; doi:10.1007/s00425-022-03899-y)
Supplement: Supplementary file 6 — Supplementary file6 (DOCX 210 KB) [file 425_2022_3899_MOESM6_ESM.docx]

Supplementary Fig. S3

Standard curve for GA_3_ and GA_55_. Standard curves were developed for the 3-day and 4-day incubation periods at 20 °C for both GA_3_ and GA_55_ with distal halves of wheat grains, cv Hartog. Total α-amylase activity was determined using a modification of the Megazyme Amylazyme^TM^ (Megazyme Ltd., Bray, Ireland) assay described in the Materials and Methods section of the manuscript. OD was measured at 590nm in a Bio-Rad Benchmark Plus microplate reader (Bio-Rad Laboratories, Gladesville, NSW, Australia). Results were expressed as optical density units. Each point represents the average of four replicates measured twice. X-axis

0

2

4

6

8

10

12

14

0.0001

0.001

0.01

0.1

1

α-amylase

activity

(Absorbance

at 590nm)

GA Concentration

μ

g/mL (log

10

)

GA

3

4d

GA

55

4d

GA

3

3d

GA

55

3d
