# Supplementary material for: Gibberellins in developing wheat grains and their relationship to late maturity α-amylase (LMA)
Source: Planta. 2022 May 6;255(6):119. doi: 10.1007/s00425-022-03899-y (PMC9076747; doi:10.1007/s00425-022-03899-y)
Supplement: Supplementary file 7 — Supplementary file7 (PPTX 42 KB) [file 425_2022_3899_MOESM7_ESM.pptx]

## Slide 1
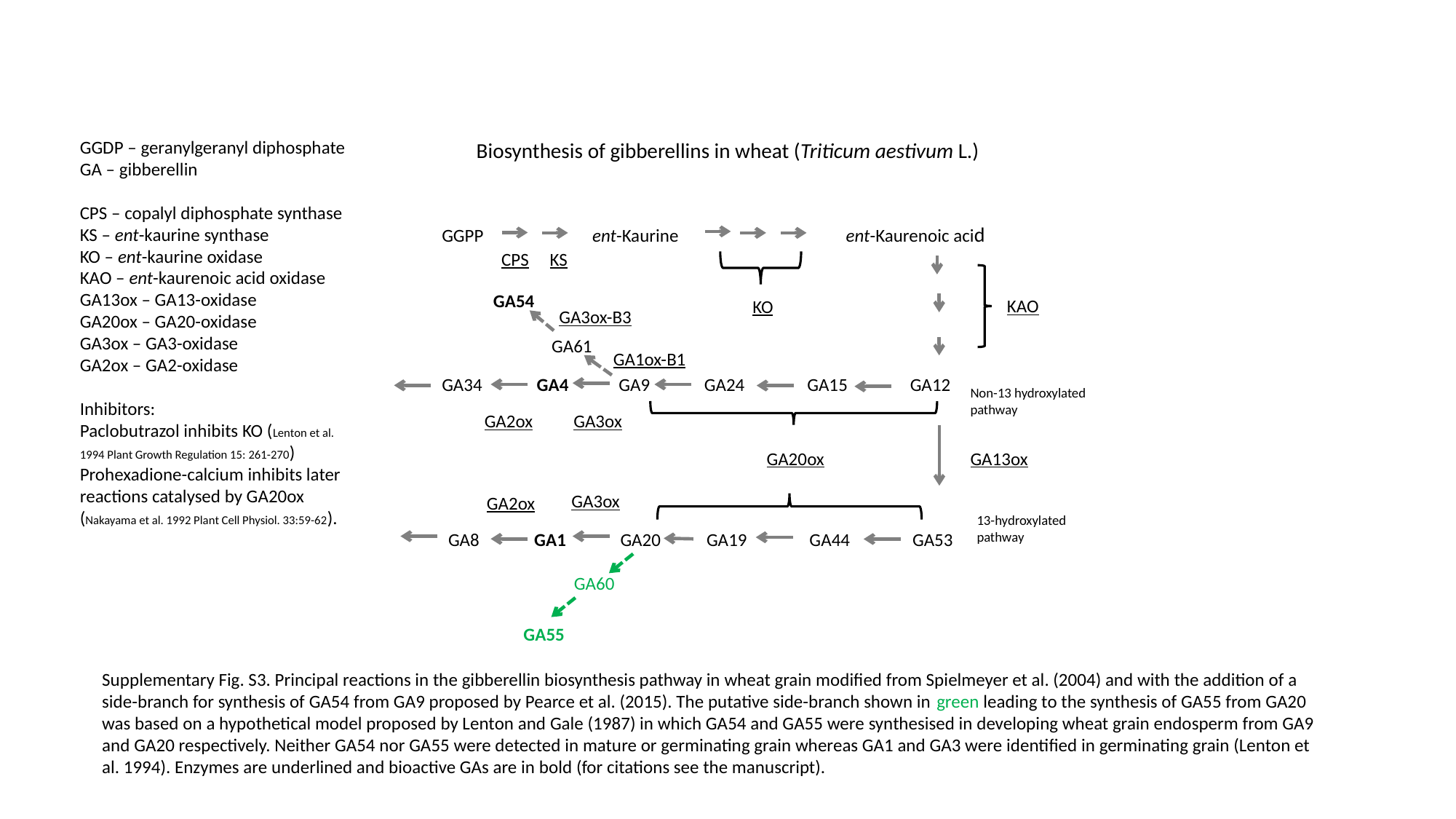

GGDP – geranylgeranyl diphosphate
GA – gibberellin
CPS – copalyl diphosphate synthase
KS – ent-kaurine synthase
KO – ent-kaurine oxidase
KAO – ent-kaurenoic acid oxidase
GA13ox – GA13-oxidase
GA20ox – GA20-oxidase
GA3ox – GA3-oxidase
GA2ox – GA2-oxidase
Inhibitors:
Paclobutrazol inhibits KO (Lenton et al. 1994 Plant Growth Regulation 15: 261-270)
Prohexadione-calcium inhibits later reactions catalysed by GA20ox (Nakayama et al. 1992 Plant Cell Physiol. 33:59-62).
Biosynthesis of gibberellins in wheat (Triticum aestivum L.)
GGPP ent-Kaurine ent-Kaurenoic acid
CPS KS
GA54
KAO
KO
GA3ox-B3
GA61
GA1ox-B1
GA34 GA4 GA9 GA24 GA15 GA12
Non-13 hydroxylated pathway
GA2ox
GA3ox
GA20ox
GA13ox
GA3ox
GA2ox
13-hydroxylated pathway
GA8 GA1 GA20 GA19 GA44 GA53
GA60
GA55
Supplementary Fig. S3. Principal reactions in the gibberellin biosynthesis pathway in wheat grain modified from Spielmeyer et al. (2004) and with the addition of a side-branch for synthesis of GA54 from GA9 proposed by Pearce et al. (2015). The putative side-branch shown in green leading to the synthesis of GA55 from GA20 was based on a hypothetical model proposed by Lenton and Gale (1987) in which GA54 and GA55 were synthesised in developing wheat grain endosperm from GA9 and GA20 respectively. Neither GA54 nor GA55 were detected in mature or germinating grain whereas GA1 and GA3 were identified in germinating grain (Lenton et al. 1994). Enzymes are underlined and bioactive GAs are in bold (for citations see the manuscript).
